# THE PSYCHOSOCIAL EFFECTS OF EXPRESSIVE ARTS THERAPY ON PATIENTS WITH AGE-RELATED MACULAR DEGENERATION

**DOI:** 10.1093/geroni/igae098.3508

**Published:** 2024-12-31

**Authors:** Rainbow Tin Hung Ho, Temmy L T Lo, Wai Chi Chan, Allen M Y Cheong, Qing Li, Adrian H Y Wan

**Affiliations:** The University of Hong Kong, Hong Kong, China (People’s Republic); The University of Hong Kong, Hong Kong, China (People’s Republic); The University of Hong Kong, Hong Kong, China (People’s Republic); The Hong Kong Polytechnic University, Hong Kong, Hong Kong; Grantham Hospital, Grantham Hospital, Hong Kong; The University of Hong Kong, Hong Kong, China (People’s Republic)

## Abstract

Age-related macular degeneration (AMD) is the leading cause of central vision loss in older adults. With irreversible vision loss, patients with AMD also experience psychosocial distress, which is given less attention. This study aims to evaluate the psychosocial effects of Expressive arts therapy (EXAT) on patients with AMD. 33 participants aged 51–74 were randomized into the EXAT (N=20) and control (N=13) group. Participants in the EXAT group received eight weekly 1.5-hour sessions, whereas participants in the control group maintained their usual routine. The psychosocial condition of participants was assessed at baseline (T0) and post-intervention for the EXAT group or 8-week follow-up for the control group (T1). Mann-Whitney U Tests revealed that participants in the EXAT group had significantly higher social support and quality of life in mental health than those in the control group at T0 (Z = -3.231 - -2.093). It also showed that the EXAT group had significantly higher resilience, depressive symptoms, psychosocial adaptation, social support, and quality of life in the ocular pain aspect compared to the control group at T1 (Z = -2.838 - -2.157). Wilcoxon Signed-Ranks Tests found significant improvement in the EXAT group in resilience, psychosocial adaptation in anxiety/depression aspect, and quality of life in role difficulties aspect (Z = -2.255 - -2.017), with no significant changes in the control group across the two times. The results demonstrated that EXAT can potentially enhance patients’ psychosocial well-being with AMD. A larger sample size and follow-up assessments are needed for further investigation.

